# Evaluation of a WASH/MNCH targeted primary health care program in Uganda; a mixed methods study

**DOI:** 10.1080/21642850.2023.2185245

**Published:** 2023-03-13

**Authors:** Comfort Hajra Mukasa, Maureen Nankanja, Margaret Mugisa, Ojoro Valentine, Patrick Kagurusi

**Affiliations:** Programmes, Amref Health Africa in Uganda, Kampala, Uganda

**Keywords:** WASH/MNCH, integrated interventions, skilled birth attendance, neonatal sepsis, Uganda

## Abstract

**Background:**

Evidence on the impact of Maternal Newborn and child health (MNCH) and Water, Sanitation and Hygiene (WASH) interventions on skilled birth attendance (SBA) and neonatal sepsis remains unclear. We conducted this study in Amuru district to generate evidence.

**Methods:**

A before-and-after study design was conducted. Interventions implemented included; training of HCWs, construction of WASH facilities, and health education of communities. A digitized structured questionnaire was used to obtain data on ANC and SBA, WASH practices and prevalence of pneumonia and diarrhea among 466 expectant mothers and caretakers of under-fives at baseline, midterm and endline. Data on sepsis incidence, ANC, SBA and WASH status was obtained from six healthcare facilities. A total of 12 KIIs and 12 FGDs were conducted. Data were analyzed using STATA 15. Two sample tests of proportions were used to compare findings at baseline and endline. Qualitative data were analyzed using thematic content analysis.

**Results:**

The number of women delivering at HCFs significantly increased from 41.4% to 63.0% (*p* < .0001). Incidence of neonatal sepsis reduced from 0.6% to 0.2% (*p* = .0687), although the difference was not significant. Community-level findings also indicated a decline in cases of water-borne illnesses; cases of dysentery decreased from 10.0% to 0.6%, cases of cholera decreased from 8.9% to 1.9% at endline, cases of typhoid decreased from 26.5% to 12.7% at endline.

**Conclusion:**

This study revealed that integrated WASH/MNCH interventions can significantly increase ANC and SBA, reduce incidences of neonatal sepsis, diarrhea, pneumonia, and other related diseases and improve WASH practices in communities. Significant improvements in WASH/IPC and the capacity of HCWs to deliver safe MNCH services are realized.

## Background

Maternal and neonatal mortality remain serious public health concerns, especially in low- and middle-income countries (LMICs). In 2017, over 295,000 women worldwide died from preventable causes related to pregnancy and childbirth (WHO, 2022b). Recently, under-five mortality has substantially reduced, but neonatal mortality remains stagnated and high (Sharrow et al., 2022; UNICEF, 2021). Approximately 2.4 million neonatal deaths occurred in 2020 (Asiimwe et al., 2019; UNICEF, 2021). Sub-Saharan Africa (SSA) is disproportionately affected and carries the largest share of maternal (66%) and neonatal deaths (27 deaths per 1000 live births) compared to other regions (UNICEF, 2021; WHO, 2022a, WHO, 2022b). Similarly, in Uganda, recent estimates point to high maternal and neonatal mortality rates of 375/100,000 live births and 19/1000 live births, respectively (World Bank, 2022).

Nearly three-quarters of all maternal deaths are caused by hemorrhage, infections, pre-eclampsia and eclampsia, and complications from delivery and unsafe abortion (WHO, 2022b). Sepsis, pneumonia, tetanus, diarrhea, prematurity and birth asphyxia account for the majority of neonatal deaths (Kananura et al., 2016). Particularly, sepsis is responsible for 225,000 neonatal deaths globally per annum (Tumuhamye et al., 2020). Poor maternal and neonatal outcomes stem from a lack of quality care during pregnancy and childbirth, especially in LMICs (Dahab & Sakellariou, 2020) like Uganda. In addition, complications after childbirth such as infections have partly been attributed to poor hygiene and sanitation practices (Campbell et al., 2015; Dahab & Sakellariou, 2020; WHO, 2019).

Existing evidence shows that majority of the maternal and neonatal deaths can be averted with improved access to and utilization of SBA at all levels (Stenberg et al., 2014; Wafula et al., 2021; Wilunda et al., 2015), and improved access to WASH at the healthcare facilities (HCFs) and communities (Benova, Cumming, and Campbell, 2014;Campbell et al., 2015). Adequate WASH services are a prerequisite for the delivery of quality MNCH services including hygienic birth practices such as hand washing by birth attendants, cleaning the maternal perineum, use of a clean birth surface, cutting and tying of the cord. WASH interventions can reduce neonatal sepsis deaths, neonatal tetanus deaths and maternal sepsis deaths (Blencowe et al., 2011; Campbell et al., 2015; Pollard et al., 2013; WHO and UNICEF, 2021). Despite this, many HCFs and communities in LMICs lack basic WASH (WHO and UNICEF, 2021). In addition, the Uganda Demographic Health survey estimated that only 74% of births are assisted by a skilled attendant, which still falls short of the 90% target (Munabi-Babigumira et al., 2019; UDHS, 2016). These challenges are however more significant in some regions than others. Amuru district, located in the post-war northern region of Uganda, still faces a notable burden of hemorrhage, eclampsia and sepsis as a result of poor ANC and postnatal care, and low levels of SBA. According to Annual Health Sector Performance Report (AHSPR, 2014) (MOH, 2014), Amuru district stood at 17.9% deliveries at facility level, first ANC attendance at 52.2% while the fourth ANC attendance at 32.9%. Additionally, with no referral health center, the district reports high numbers of new-born deaths, attributed to limited coverage of new-born skilled care, high-risk of infections due to poor hygiene, and poor WASH indicators, with 28% Open defecation (OD). Moreover, the district is ranked among the top 10 hard to reach areas in the country which impacts service delivery and support from partners (MOH, 2015).

Owing to the public health significance of maternal and neonatal mortality, countries adopted various strategies and committed to the UN Sustainable Development Goals (SDGs). SDG 3.1, particularly focuses global attention on the reduction of the maternal mortality ratio (MMR) to fewer than 70/100,000 live births by 2030 (Callister & Edwards, 2017). Furthermore, SDG 3.2 includes an ambitious target of ‘Ending preventable deaths of newborns and children under 5 years of age by 2030’ (WHO and UNICEF, 2020). Progress has been made over the years and maternal mortality has dropped substantially. However, the reduction is less than half the 6.4% annual rate needed to achieve the SDG global goal of 70 maternal deaths per 100,000 live births (UNICEF, 2021). In addition, the gains in the reduction of neonatal mortality have been slow over the years (UNICEF, 2021) and remain stagnated in countries like Uganda (UDHS, 2016).

Meeting national and global commitments needs a combination of strategies, including integration of MNCH and WASH interventions. Whereas there is consensus on the importance of WASH/MNCH for the prevention of maternal and neonatal mortality (Benova, Cumming, Gordon, et al., 2014; Delele et al., 2021), estimating the size of the effect has been more challenging (Benova, Cumming, Gordon, et al., 2014). Furthermore, there are few reports addressing the potential benefits and challenges of integrated programming, despite the conceivable linkages between WASH and MNCH services. This study therefore, generated evidence to inform appropriate interventions.

## Methodology

### Study design and setting

A before and after study design comparing non-randomly assigned intervention and control groups was conducted. Cross-sectional surveys were conducted at baseline (pretest), midline and endline (posttest). The pre-intervention period acted as the control. This study was conducted in Amuru District, located in post-war Northern Uganda. The District is made up of 4 Sub-counties and 1 Town Council, 32 parishes and 63 villages, with a total population of 186,696 people (Amuru District Local Government, 2021; UBOS, 2014). Despite the banning of TBAs, the government cannot enforce the ban and the practice remains common in the region due to the inconsistencies and gaps in the main healthcare system (Monitor, 2021).

### Interventions

Interventions were delivered through the Total Health Project (THP) which was implemented by Amref Health Africa in partnership with the Netherlands government. The project integrated WASH and MNCH interventions in four sub-counties including; Amuru, Pabbo, Attiak and Lamogi, from which six parishes and six HCFs were purposively selected to receive the interventions between 2019 and 2021. These HCFs and communities were identified and selected under the guidance of the District Health Team (DHT) for having the highest WASH and MNCH needs. The package of interventions implemented included the following:

At the HCFs

1. On-job training and mentorship of healthcare workers (HCWs) on current MNCH services. The training was conducted by THP staff among HCWs especially those who offer MNCH services to strengthen their capacity to offer clean, safe and effective MNCH services.
2. Facilitating integrated outreach services for HCWs serving in the high-volume facilities through transportation of HCWs to offer MNCH services.
3. Installing water systems to provide running water in sluice rooms and maternity wards in the six HCFs.
4. Constructing lined pit latrines. Abandoning filled-up latrines is not a sustainable measure, therefore the project supported the HCFs by constructing lined pit latrines that can be emptied and reused.
5. Constructing post-delivery washrooms at health centers to provide space for recovering mothers post-delivery and improve privacy.

At the community level

1. Training community artisans to construct low-cost appropriate improved latrines. With the help of the local leadership, community artisans in each parish were identified and trained on how to construct improved latrines taking into account the comfort, safety, privacy and maintenance aspects.
2. Training Village Health Teams (VHTs) to promote door-to-door health education on sanitation and hygiene practices.
3. Home improvement campaigns; Triggering of communities with high rates of OD to improve their sanitation through Community Led Total Sanitation (CLTS). Additionally, environmental and personal hygiene were promoted as well as advocacy for SBA and ANC.

### Study population and eligibility criteria

The study population included pregnant women, caretakers of children under five years, HCWs, HCF in charge and focal persons, VHTs and the DHT including health assistants and health inspectors. The DHT members were interviewed as key informants while pregnant women and caretakers of under-fives were involved in the household surveys and FGDs. Only expectant mothers or caretakers of children under-five aged 18 years and above and had resided in the project areas for at least six months prior were included in the study. Households with an expectant mother or caretaker of under-five/s who was critically ill or not in the right mental state to respond to questions were excluded.

### Data collection methods and tools

A household survey was conducted among expectant mothers, mothers/caretakers of under-fives using a digitized structured questionnaire. The tool elicited data on; the socio-demographic characteristics, history of ANC attendance, SBA, WASH practices and prevalence of pneumonia and diarrhea at the community level. Health facility surveys were also conducted using digitized structured questionnaires to elicit data on; the incidence of sepsis, diarrhea and pneumonia at the health facility, ANC attendance, SBA, and WASH status of the facility. These were recorded through a review of registers in the facilities. The RAs identified the respondents, sought permission and consent, and administered the questionnaires. An observational checklist was used to verify the status of WASH and MNCH in the households and HCFs. FGDs and KIIs were conducted using respective guides to explain the changes in key indicators. Each FGD consisted of homogeneous groups of between 8 and 12 women. A moderator and a note-taker conducted the interviews.

### Sample size considerations and sampling technique

#### Quantitative component

##### Household survey sample size

The household sample size was calculated using the formula; *n* = *N*/[1 + *N* (*e*^2^)], where: *e* = 0.046 (4.6%) is the desired 95.4% level of precision, *N* = Beneficiary households are estimated at 35,921 (total number of Households in targeted sub-counties, *n* = required sample size). A sample size of 466 pregnant women/children caretakers was targeted in each of the surveys.

##### Health center survey sample

Six high-volume HCFs were selected for the interventions and surveys. For each HCF, two respondents were purposively selected to respond to the MNCH and WASH questionnaire based on their knowledge and experience of the implementation of WASH and MNCH activities. The MNCH questionnaire was responded to by either the facility in charge or MNCH focal person depending on the availability, while the WASH questionnaire was responded to by the facility in charge. The health inspector/assistant responded to the WASH questions.

#### Qualitative component

A total of 12 KIIs and 12 FGDs were conducted. The sample sizes for both KIIs and FGDs were determined by thematic saturation. KIs included; CAO/ District Planner/Secretary for social services; Water User committee/Health Unit Management committee member, VHTs, Project staff and in-charge of HCFs. FGD participants included women/caretakers of under-fives and expectant mothers.

#### Sampling technique

The HCFs and parishes were purposively selected with the guidance of the DHT for having the highest WASH and MNCH challenges in the district. The 466 expectant mothers/children caretakers were selected proportionate to size for each sub-county, parish and village. The village sample was further divided by 15 (minimum number of households per village) to yield sub-villages to be sampled in each village. In each sub-village, an average of 15 households with expectant mothers/mothers of children less than five years was selected using systematic sampling techniques. Where a selected household did not have an expectant mother/child under five, it was skipped. In case the sampled household had more than one mother with a child under five or more than one child under five, only one was chosen using simple random sampling. KIs that had worked in the project area for at least one year and interfaced with the intervention were purposively selected for the study.

### Variables and measurement

The key outcome variables included ANC attendance, SBA, incidence of sepsis, the prevalence of pneumonia, diarrhea and other related diseases and sanitation and hygiene practices of the mothers. SBA was defined as the proportion of women who delivered from the project HCFs. The number of women attending ANC in each facility was also recorded and compared across the surveys. Cases of sepsis were also recorded from health facility records and compared across the surveys in the different sub-counties. Furthermore, the prevalence of pneumonia and diarrhea among children under five was measured by asking the mothers/caretakers if their household had experienced each of those infections in the past two weeks preceding the survey. We also assessed sanitation by establishing latrine coverage and status of OD in the target communities. Hygiene practices were measured by asking whether mothers/caretakers exhibited certain practices of interest as detailed in the results.

### Data management and analysis

#### Quantitative data analysis

Data were checked for any errors and inconsistencies and uploaded onto a common server daily. Data were exported to Microsoft Office Excel for cleaning then imported into STATA v15 for analysis. Continuous variables were presented as means with standard deviations. Frequencies and percentages were used for the categorical variables. Categorical characteristics were compared across surveys. Two sample test of proportions was used to compare findings at baseline and endline. A two-sided statistical test was used with 95% confidence interval.

#### Qualitative data analysis

For qualitative data, audio recordings were transcribed verbatim and then translated into English by a team of experts. Transcripts were read thoroughly to gain understanding of the context of each KI and FGD. We then developed codes and codebook definitions based on the study objectives. The codebook was discussed and agreed upon by the qualitative analysts. Coding of the transcripts was done using Atlas ti to ease analysis. Since the transcripts were reread several times, some new codes were created and added to the existing list. Similar codes were grouped to form sub-themes which were merged into themes. The authors reviewed and agreed upon the codes, sub-themes and themes. Themes were summarized using a data master sheet.

### Quality control and assurance

The RAs were trained on data collection procedures and research ethics before start of the study. RAs were supervised by the principal investigators. Data were checked daily by the principal investigators for any errors or inconsistencies.

### Ethical statement

Ethical approval was obtained from the Mildmay Uganda Research Ethics Committee (MUREC) (MUREC-2021-72). The study was also registered with the Uganda National Council for Science and Technology. Administrative clearance was sought from the district authorities and the local leaders in the implementation parishes and villages. Written informed consent was sought from the respondents prior to all interviews. We ensured that confidentiality was maintained through storing data on password-protected computers and restricting data to only members of the study team. Participants were assured that participation was voluntary and that they did not have to answer any questions that made them feel uncomfortable.

## Results

### Socio-demographic characteristics of the respondents for the household survey

About 784 women (469 at baseline and 315 at endline evaluation) were included in the analysis. The average age of the respondents was 29 (±7.7) years at baseline and 29.6 (±7.7) at endline. At baseline, about 35.8% of the respondents had no formal education compared to 15.0% at endline, and 86.5% were married/cohabiting compared to 98.7% at endline (Table 1).

**Table 1.** Socio-demographic characteristics of the household members in Amuru district, Uganda.

Variable	Attribute	Baseline (*n* = 469)	Endline (315)
Age	18–24	41.0	14.0
	25–32	30.5	34.0
	33–39	15.8	31.0
	40+	12.7	21.0
Education level	No formal education	35.8	16.0
	Primary completed	11.5	7.0
	Primary not completed	43.3	71.0
	Ordinary level completed	2.2	2.0
	Ordinary level not completed	7.0	4.0
	Advanced level not completed	0.2	0.0
	Tertiary	0.4	1.0
Religion	Catholic	77.8	71.4
	Anglican	12.8	13.9
	Pentecostal	7.9	12.3
	Muslim	0.6	1.8
	Adventists	0.4	0.0
	Traditionalists	0.5	0.6
Marital status	Single	3.4	0.0
	Married/cohabiting	87.0	98.8
	Separated	5.5	0.6
	Divorced	4.1	0.6
Tribe	Acholi	90.2	93.9
	Madi	4.9	4.8
	Langi	3.2	0.3
	Other*	1.7	1

*Others; Alur, Lugbar, Baganda, Basoga.

### Effect of the integration of WASH and MNCH interventions on sanitation and hygiene practices among households in Amuru district

There was significant improvement in sanitation and hygiene practices at the household levels. The highest percentage point increase in households where there was no observed OD was noted in Amuru (50.3%), Pabbo (49.9%), Atiak (44.8%) and Lamogi (36.1%) (*p* < .05). There was an increase in the percentage of households with sanitation facilities from 70.9% to 81.3%. Overall, there was significant improvement in the following from baseline to endline: Washing hands with soap before feeding a child or preparing food from 41% to 59% (*p* < .001), washing hands with soap after using latrine improved from 31.8% to 59.0% (*p* < .001), and cleaning the latrines every day improved from 29.8% to 60% (*p* < .001) (Table 2).

**Table 2.** Sanitation and hygiene at baseline and endline evaluation in Amuru district, Uganda.

Indicator	Baseline (*n* = 469) %					Endline (*n* = 315) %					*P*-value
	Amuru	Atiak	Lamogi	Pabbo	Total	Amuru	Atiak	Lamogi	Pabbo	Total	
% HHs washing hands with soap before feeding a child or preparing food	40.0	30.8	43.1	50.9	41.0	53.0	47.0	67.0	69.0	59.0	<.001*
% HHs washing hands with soap after using Latrine	24.2	24.8	31.0	49.1	31.8	57.0	53.0	56.0	69.0	59.0	<.001*
% HHs cleaning the latrines every day	26.7	30.8	29.3	33.0	29.8	47.0	63.0	61.0	69.0	60.0	<.001*
% HHs keeping water and soap for washing hands at latrine	16.7	14.5	15.5	19.8	16.6	53.0	33.0	28.0	56.0	43.0	<.001*
% clearing bushes around the home	49.2	53.0	52.6	41.5	49.2	67.0	63.0	44.0	67.0	60.0	.0030
% avoiding stagnant water around the home	16.7	11.1	35.3	13.2	19.2	30.0	20.0	17.0	31.0	24.0	.1065
% HHs keeping the children’s cloths clean	39.2	47.9	43.1	38.7	42.3	60.0	50.0	33.0	31.0	43.0	.8459
% keeping utensils clean	43.30	45.3	39.7	46.2	43.6	80.0	63.0	44.0	69.0	64.0	<.001*
% disposing of children’s feces in the latrine	18.2	19.3	21.7	20.3	20.3	57.0	20.0	39.0	28.0	36.0	<.001*
% HHs having a rack for utensils in the home	5.8	7.7	9.5	8.5	7.8	23.0	17.0	17.0	28.0	21.0	<.001*
% HHs with latrines	62.9	67.5	54.2	81.3	70.9	81.2	89.4	58.3	80.9	81.3	.001*
% open defecation-free villages	45.2	49.3	59.3	55.1	54.2	92.8	94.1	95.4	100.0	95.7	<.001*

* p-Value less than 5%.

### Effect of the integration of WASH and MNCH interventions on ANC and SBA in Amuru district

**Figure 1.**
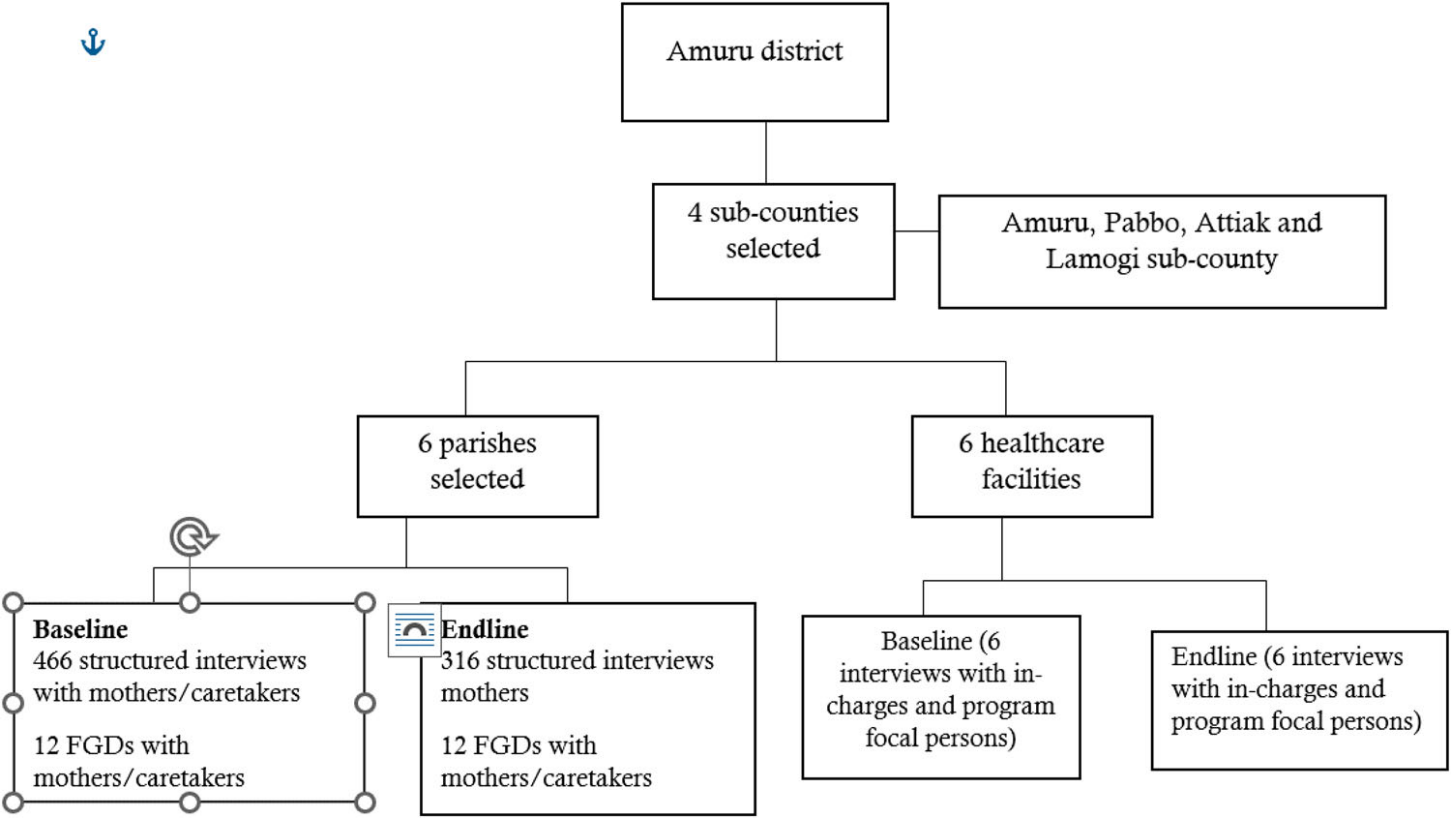
Comparison of the number of pregnant women attending the fourth ANC visit at baseline and endline in Amuru district.

There was a significant increase in the percentage of deliveries at Olwal HC from 2.4% at baseline to 11% at endline (*p* = <.001); from 4.2% to 12% at Kaladima HC (*p* = <.001); from 3.0% to 6% at Atiak HC (*p* = <.001); and 5.1% to 15% at Pabbo HC (*p* = <.001) (Table 3). The study showed a significant improvement in the percentage of pregnant women who attended their fourth ANC visit in the last one year preceding the survey (Figure 1).

**Table 3.** SBA compared between baseline, midterm review and endline evaluation in Amuru district.

Health Center	Baseline evaluation			Midterm review			Endline evaluation			*P*-value
	Deliveries in HC	Pregnant women and women of reproductive age (14–49)	% Deliveries	Deliveries in HC	Total of pregnant women and women of reproductive age (14–49)	% Deliveries	Deliveries in HC	Total of pregnant women and women of reproductive age (14–49)	% Deliveries	
Olwal	120	5019	2.4	239	782	7.5	336	3169	11.0	<.001*
Bibia	233	1983	11.7	166	586	7.1	209	2344	9.0	<.001*
Kaladima	114	2683	4.2	74	610	3.0	286	2465	12.0	<.001*
Labongogali	280	1868	15.0	257	580	9.6	263	2668	10.0	<.001*
Atiak	219	7375	3.0	140	748	4.1	263	4201	6.0	<.001*
Pabbo	291	5668	5.1	74	723	2.5	430	2942	15.0	<.001*
Total		24596	41.4					17789	63.0	<.001*

* *p*-Value less than 5%.

### Effect of the integration of WASH and MNCH interventions on cases of neonatal sepsis, pneumonia and diarrheal diseases in Amuru district

**Figure 2.**
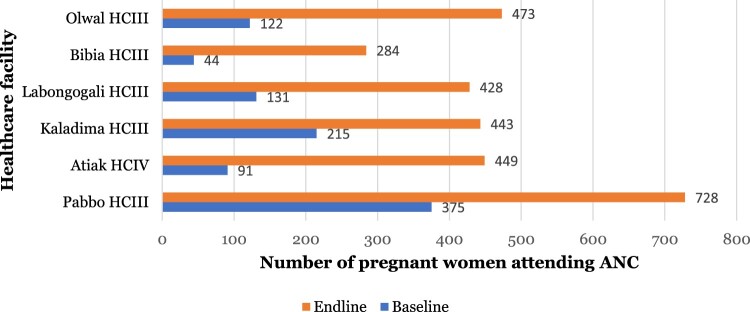
Prevalence of water-borne illnesses in targeted households in Amuru district two weeks preceding the survey.

At HCF level, there was also significant reduction in neonatal sepsis (Table 4) and in cases of diarrhea, pneumonia, and other related diseases in Atiak, Lamogi, Amuru and Pabbo sub-counties (Table 5). There was also a decline in cases of water-borne illnesses at household level; cases of dysentery decreased from 10.0% to 0.6% (*p* < .001), cases of cholera decreased from 8.9% to 1.9% (*p* = .001), and cases of typhoid decreased from 26.5% to 12.7% (*p* < .001) (Figure 2).

**Table 4.** Shows the number of neonates presented and cases of sepsis recorded at project health facilities that were supported by the THP.

Health Facility	Baseline				Mid-Term Review				Endline			
	Neonates	Male with sepsis	Female with sepsis	Total	Neonates	Male with sepsis	Female with sepsis	Total	Neonates	Male with sepsis	Female with sepsis	Total
Olwal HC III	120	0	0	0	197	0	0	0	336	0	0	0
Bibia HC III	233	2	3	5	122	0	0	0	209	1	0	1
Labongogali HC II	279	0	0	0	334	0	0	0	321	0	0	0
Kaladima HC III	114	1	0	1	123	2	1	3	286	0	0	0
Atiak HC IV	219	0	0	0	125	1	0	1	264	2	0	2
Pabbo HC III	291	1	0	1	229	0	0	0	430	0	0	0
All HCs	1256	4	3	7	1,097	3	1	4	1,845	3	0	3
				0.60%				0.40%				0.20%

**Table 5.** Cases of diarrhea, pneumonia, and other related diseases in the targeted communities of Amuru district in the previous six months preceding the surveys.

Sub-county	Baseline			Endline		
	Diarrhea	Pneumonia	Others	Diarrhea	Pneumonia	Others
Amuru	25	58	6	3	0	3
Atiak	18	64	3	6	3	2
Lamogi	20	62	8	2	2	5
Pabbo	23	66	6	2	1	1

### Qualitative findings on the effect of the interventions on the status of WASH and MNCH

Respondents affirmed that there was significant improvement in the status of WASH and MNCH in the project areas. Table 6 summarizes the themes, sub-themes and codes that arose.

**Table 6.** Effect of the intervention on the status of WASH and MNCH in Amuru district.

Themes	Sub-themes	Codes
Effect of the intervention on the status of WASH	Effect of the intervention on WASH in households	Increase in latrine coverage
		Reduction in open defecation
		Improvement in hygiene practices
		Improvement in access to water
	Effect of the intervention on WASH in targeted healthcare facilities	Improvement in WASH in the maternity wards at the target healthcare facilities
		Reduction in cases of diarrheal diseases
Effect of the intervention on MNCH	Effect on healthcare worker capacity regarding delivery of MNCH services	Increase in availability of water in maternity wards
		Improvement in hygiene status of maternity wards in intervention facilities
		Improvement in the capacity of health workers to deliver clean and safe MNCH services in Amuru District
	Effect on the incidence of MNCH conditions	Reduction in cases of sepsis
		Reduction in cases of pneumonia in neonates

### Increase in latrine coverage and reduction in the prevalence of open defection at baseline and endline

Respondents highlighted that there was a reduction in the practice of OD in the project areas. Seventeen, 63% (17/27) of the villages were declared free of OD at the endline evaluation.

In the targeted households, most families that used to ease themselves in the bush have Latrines in their homes now. Currently, we have registered up to 17 villages that are OD Free. This is a tremendous change because it has not been easy for local governments to achieve ODF. And with support from Amref, some villages were declared free and they have maintained that condition. They have not gone back to OD (OD) as it used to be. However, in some areas where AMREF has not visited, the practice still exists. Of course the target was 27 villages. We achieved 17. There was some more that should have emerged but since the project ended, we shall try to work around. We shall continue as local government to have them declared … . (KII 3)

AMREF has been sensitizing communities … this has contributed to a great improvement in hygiene in our families. Community members have latrines. There is no OD. AMREF worked with the local leaders to enforce hygiene in the community as a strategy for promoting hygiene. All men with more than one wife were required to construct a latrine for each wife. Ten community members from the village were selected to carry out monitoring of hygiene situation. (FGD 2)

### Improvement in hygiene practices

Respondents also expressed improvements in hygiene practices among households. Particularly, KIs mentioned increase in hand washing among households in the intervention areas.

There has been significant improvement in HH among households with mothers and children under 5 and the general community members because all households in the targeted villages of THP interventions have been using hand washing facilities. There has been construction of new tippy taps and maintenance of broken ones in many of the homes around … . (KII 5)

### Increased access to water in the project areas

Access to water became easy in the project areas. Boreholes were rehabilitated and springs were protected, which increased access. The communities further organized saving schemes to ensure sustainability of the water sources beyond the project timeline.

AMREF supported a total of 10 water sources including rehabilitation of 5 Boreholes and protected 5 springs and ensured the formation of water user committee for all the water sources. We also encouraged them to save. There was a water source committee for some Boreholes in Acodo village in Mutema Amuru Sub County who saved up to two million Uganda shillings for sustaining the Borehole. This improved the water status. (KII 14)

For me I am fully convinced that whether these people (AMREF) leave, the VHTs are there to continue the work. The infrastructures they have built, even if they leave the water will be there and the community will be using it. They have set up structures; water user committee doing caretaking. It’s a recognized system or structure. Community people are overseeing it. The money being collected for maintenance of water sources is not for Amref, it’s for the community … . (KII 15)

### Improvement in WASH in the maternity wards at the target HCFs

There was an improvement in the WASH status of the project HCFs due to infrastructural developments. Respondents reported an improvement in status of WASH as a result of construction of placenta pits, provision of safe running water, and provision of medical equipment like sterilization equipment, delivery pacts and improved lighting.

… There has been a change in hygiene due to the construction of a placenta pit. Sterilization of instruments has also led to reduction in the rate of neonatal sepsis since there is a good sterilizer and the instruments were boiled in the past. This is a great change brought by AMREF  … . (KII 11)

 …  Since there was no power in the maternity ward, we used to conduct delivery at night using torches but now we have solar lights and this has been a great change. Pregnant mothers used to pump water from the borehole but now the solar-powered water pump enables water to flow direct from the tank to the maternity. This is a great change since it has improved hygiene and reduction in neonatal sepsis … . (KII 7)

### Capacity of HCWs to deliver clean and safe MNCH services in Amuru district

Qualitative findings also indicated an improvement in the capacity of HCWs concerning the delivery of clean and safe MNCH services in Amuru district. Over 25 HCWs received training on customer care, provision of basic obstetrics, antenatal care, management of maternal complications and neonatal complications during delivery like birth asphyxia and obstructed labor, which greatly improved the quality of care.

We were trained on customer care, managing complications during delivery, handling pregnant mothers, management of obstructed labour, provision of basic obstetrics and emergency care of mothers in labour, resuscitation of babies, provision of antenatal and postnatal care as well as on job coaching and mentoring using senior health practitioners from outside Amuru District. This significantly contributed to enhanced capacity of skilled HCWs to deliver clean and safe services and improvement in the quality of service delivery … . (KII 3)

… Training that took place was expected to improve the mind-set, behaviours and attitudes of midwives on how to treat mothers. It’s all known throughout this country that midwives are rude. Midwives beat mothers, midwives abuse mothers so under this project they allocated some funding … We brought some senior midwives from Kampala to talk to these midwives … so that they can improve on their attitudes toward mothers … I think it was a very important training that needs to be emphasized … there was also equipment that was given under this project, diagnostic equipment, liquid soap for service delivery. They also supported my office with support supervision, going out in the field to see what is taking place … . (KII)

### Effect of the integration of WASH and MNCH interventions on cases of neonatal sepsis, pneumonia and diarrheal diseases in Amuru district

From the qualitative findings, expressed that the interventions under the THP such as: the renovation of the maternity units, provision of water, electricity and medical equipment, tools, drugs, etc., as contributed to a reduction in neonatal sepsis.

Infrastructural development was the main reason for reducing neonatal sepsis. They connected water to the ward. The transmission mechanism behind neonatal sepsis is poor hygiene in the ward. This poor hygiene needs a lot of water for cleaning. It needs constant cleaning. When no water is when you have bacteria that cause those infections like neonatal sepsis. Those areas have been addressed by THP. I haven’t reported any new cases of neonatal sepsis. And also when I look at my statistics, neonatal cases are not seen … . (KII 1)

The situation of maternity units was very bad in terms of hygiene and sanitation and this was leading to high rate of neonatal sepsis and other infection among children and mothers before the implementation of THP by Amref Health Africa. There was no water in the maternity units and provision of running water and other supplies contributed greatly towards improvement of hygiene and sanitation in the maternity units and reduction in the rate of neonatal sepsis situation was very bad and this was causing diseases … . (FGD Sub County 1)

## Discussion

This study assessed the effect of WASH/MNCH integrated interventions on SBA, the incidence of childhood infections and WASH practices in Amuru District, Uganda. The findings indicated that integrated WASH/MNCH interventions can significantly increase ANC and SBA, reduce incidences of neonatal sepsis, diarrhea, pneumonia, and other related diseases and improve WASH practices in communities. Furthermore, there were significant improvements in WASH/IPC and the capacity of HCWs to deliver clean and safe MNCH services.

Overall, the number of women delivering at HCFs supported by the project significantly increased between baseline and endline evaluations. This achievement can be attributed to the improvement in the quality of healthcare offered at these facilities and WASH status. Improvement in the quality of healthcare can be explained by the training held with the HCWs in these facilities. The training focused on enabling HCWs to offer clean and safe MNCH services such as basic obstetrics services, ANC and management of maternal complications and complications during delivery. The training also emphasized the mindset and behavior change of the HCWs to make them more friendly and cautious towards pregnant women and mothers seeking MNCH services. Indeed, mothers who accessed maternal services at the THP-supported HCFs expressed satisfaction with the quality of antenatal care, post-natal care and general maternity services received. Quality of basic maternal care has previously been associated with SBA (Kruk et al., 2016; Peet & Okeke, 2019). In addition, the THP improved access to WASH services and renovated maternal wards in these facilities. Provision of amenities such as water, sanitation, electricity and other supplies has been linked with skilled birth attendant’s ability to offer quality care (Munabi-Babigumira et al., 2017; Mbonye & Asimwe, 2010). The effect of improved WASH status on SBA can be explained by the fact that adequate WASH facilities and IPC practices are critical for maternal and child health even in non-facility birth. Expectant mothers are also more likely to visit a health facility where they expect to find a reliable source of water, improved sanitation facilities and provisions for personal hygiene (Arowosegbe et al., 2021). The study also revealed an increase in the percentage of pregnant women who attended their fourth ANC visit. It is likely that these women continuously noticed changes in the quality of services and infrastructure which informed their choice at the time of delivery. It is also probable that these women recommended SBA to their peers. According to Nahar et al. (2022) women who utilized more than four ANC visits showed a greater tendency to use skilled birth attendants during childbirth than their counterparts. The findings altogether indicate a need to continuously improve the quality of care at HCFs in the dimensions of effectiveness, safety and responsiveness or patient-centeredness, so that the intended users opt to use them. In pursuit of Universal Health Coverage, MNCH services that are utilized are warranted.

We also noted that integrating WASH and MNCH interventions decreased the incidence of neonatal sepsis at HCFs and significantly reduced the incidences of diarrhea, pneumonia and other related diseases in the targeted communities. Neonatal sepsis is a common condition in HCFs, especially in LMICs and has been associated with poor WASH conditions. Improving WASH and IPC services through the renovation of the maternity units, construction of hygiene infrastructure, and provision of water, electricity and medical equipment at the THP-supported facilities therefore must have led to the reduction in the incidence of these infections recorded at the HCFs. This finding is supported by existing literature. To prevent infections and provide quality care, HCFs need to have a safe and accessible water supply; clean and safe sanitation facilities, hand hygiene facilities at points of care and sanitary facilities; and appropriate waste disposal systems (CDC, 2020). It has also previously been documented that energy (electricity) is an enabler of better health service delivery and universal health coverage (WHO, 2014). It is also worth noting that there were no other interventions ongoing in these facilities during this period to confound the outcomes. Therefore, it is warranted for authorities to improve access to water, sanitation services and energy in HCFs to reduce the incidence of infectious diseases.

The study indicated great improvement in sanitation and hygiene practices at the household level. Significant improvements were recorded in practices such as hand hygiene at critical moments, as well as daily cleaning of the latrines, and clearing bushes around the home, among others. These changes can be attributed to the efforts of the VHTs. The THP, through the VHTs, implemented sensitizations of community members on sanitation and hygiene to enlighten them on the nexus between their practices, the environment and the incidence of water-related diseases. Sensitizations and mindset change have been reported to positively influence WASH practices in communities (IRC, 2016; URC, 2021; USAID, 2017). The VHTs also implemented effective outreach programs to increase the construction of pit latrines and uptake of good hygiene and sanitation practices. This study indeed found an increase in latrine coverage in the supported communities. The project further rehabilitated water sources in these communities leading to easy access to safe water. It is probable and supported by literature (USAID, 2017) that the reduction in incidences of water-borne diseases, sepsis and other hygiene-related infections discussed above was in part contingent on these community interventions. It is therefore important to promote better WASH practices in communities through a combination of strategies including communication for behavior change and the necessary WASH infrastructural improvements to eliminate barriers.

## Strengths and limitations

This may be the first study that assessed the impact of the integration of MNCH and WASH interventions on MNCH outcomes in Uganda. Our study was complemented by qualitative data collection methods which provided explanations for some of the observed changes. However, the study is not free of limitations. Some of the outcomes of the study (Sanitation and hygiene practices) were assessed through self-report, and thus the study was subject to social desirability bias. We, however, minimized this by assuring participants of privacy and confidentiality of their responses. We also used a before-and-after study design which may not have taken into account confounders that are often best handled through randomization. Additionally, since the proportion of respondents in some categories of age and education level was different at baseline and endline, this may have impacted on our findings. Hence, it was difficult to confidently attribute the changes seen in the community only to the project interventions. Nonetheless, the average age of the respondents was not significantly different across the surveys with 29 (±7.7) years at baseline and 29.6 (±7.7) at endline. Furthermore, to the best of our knowledge, no other partners were present and offering similar components of the interventions within the project period. These limitations do not invalidate the findings of this study. We recommend that a more rigorous study design such as an experimental study design is used to ascertain the impact.

## Conclusion

Integrated WASH/MNCH interventions can significantly increase ANC and SBA, reduce the incidence of neonatal sepsis, diarrhea, pneumonia, and other related diseases and improve WASH practices in communities. Significant improvements in WASH/IPC in the maternity wards and the capacity of HCWs to deliver clean and safe MNCH services can also be realized. We, therefore, recommend the integration of WASH/MNCH interventions for projects aimed at improving quality of care, SBA, reduction of childhood infections and improvement in WASH practices.

## Data Availability

The data used for this manuscript is available from the corresponding author on reasonable request.
